# Advantages of Using 3D Spheroid Culture Systems in Toxicological and Pharmacological Assessment for Osteogenesis Research

**DOI:** 10.3390/ijms25052512

**Published:** 2024-02-21

**Authors:** Chawon Yun, Sou Hyun Kim, Kyung Mok Kim, Min Hye Yang, Mi Ran Byun, Joung-Hee Kim, Doyoung Kwon, Huyen T. M. Pham, Hyo-Sop Kim, Jae-Ho Kim, Young-Suk Jung

**Affiliations:** 1 Department of Pharmacy, Research Institute for Drug Development, College of Pharmacy, Pusan National University, Busan 46241, Republic of Korea; beekey@pusan.ac.kr (C.Y.); souhyun@pusan.ac.kr (S.H.K.);; 2 College of Pharmacy, Daegu Catholic University, Gyeongsan 38430, Republic of Korea; 3 Department of Medical Beauty Care, Dongguk University Wise, Gyeongju 38066, Republic of Korea; 4 Jeju Research Institute of Pharmaceutical Sciences, College of Pharmacy, Jeju National University, Jeju 63243, Republic of Korea; kwondoy@jejunu.ac.kr; 5 Department of Molecular Science and Technology, Ajou University, Suwon 16499, Republic of Koreajhkim@ajou.ac.kr (J.-H.K.)

**Keywords:** osteogenic differentiation, 3D culture, bone regeneration, toxicological and pharmacological assessment, bone homeostasis

## Abstract

Bone differentiation is crucial for skeletal development and maintenance. Its dysfunction can cause various pathological conditions such as rickets, osteoporosis, osteogenesis imperfecta, or Paget’s disease. Although traditional two-dimensional cell culture systems have contributed significantly to our understanding of bone biology, they fail to replicate the intricate biotic environment of bone tissue. Three-dimensional (3D) spheroid cell cultures have gained widespread popularity for addressing bone defects. This review highlights the advantages of employing 3D culture systems to investigate bone differentiation. It highlights their capacity to mimic the complex in vivo environment and crucial cellular interactions pivotal to bone homeostasis. The exploration of 3D culture models in bone research offers enhanced physiological relevance, improved predictive capabilities, and reduced reliance on animal models, which have contributed to the advancement of safer and more effective strategies for drug development. Studies have highlighted the transformative potential of 3D culture systems for expanding our understanding of bone biology and developing targeted therapeutic interventions for bone-related disorders. This review explores how 3D culture systems have demonstrated promise in unraveling the intricate mechanisms governing bone homeostasis and responses to pharmacological agents.

## 1. Introduction

Research in bone health is imperative, catering to the well-being of young and old populations. The significance of understanding bone development in young individuals lies in achieving optimal bone mass, directly influencing lifelong bone strength, and mitigating the risk of osteoporosis [1]. Dysregulation of bone homeostasis during adolescence contributes to impaired bone growth, heightened fracture risks, and the onset of chronic diseases. The increase in the aging population has resulted in a rise in age-related diseases, notably those pertaining to bones [2]. Conditions like osteoporosis and fractures not only impose significant societal and healthcare burdens but also compromise the independence and well-being of older persons, underscoring the urgent need for advancements in bone health research.

Despite the strides made in bone health research, gaps and limitations exist, particularly within the context of cell culture models. While valuable, traditional two-dimensional (2D) cell cultures are constrained in accurately representing the intricate cellular environments essential for understanding bone biology [3,4]. The advent of three-dimensional (3D) cell culture models has marked a paradigm shift in bone research, surmounting the limitations of 2D cultures by providing a more faithful depiction of the physiological characteristics of bones. These 3D models excel at capturing multidimensional cell interactions and mechanical forces, thus offering a superior platform for studying bone biology [5,6].

In traditional 2D cultures, cells grow into monolayers in culture flasks or flat Petri dishes attached to the plastic surface [7]. By contrast, 3D spheroids are cellular structures formed by aggregating cells into spherical clusters, mimicking the natural cellular organization found in tissues. In bone tissue engineering, they offer distinct advantages over scaffold-based approaches [8]. The formation of spheroids is achieved by selecting the appropriate cell type and culturing it until the desired fusion. Then, spheroid formation is induced using methods like hanging drop or non-adherent surfaces. The spheroid formed in this way is transferred to a dedicated culture vessel with appropriate medium and supplements, and then experiments are conducted while monitoring the shape, molecular assays, and function of the spheroid. This method enables the creation of physiologically relevant 3D models, vital for drug testing, disease modeling, and tissue engineering, advancing biomedical research and therapeutic development [9].

Recently, there has been an increasing focus on scaffold-based bone research, as it enables and encourages the attachment and proliferation of osteo-inducible cells on the scaffold surfaces [10]. However, 3D spheroids offer several advantages over scaffold-based approaches. For instance, 3D spheroids more accurately mimic the natural microenvironment of bone tissue, thereby promoting cell–cell interactions and the deposition of extracellular matrix, leading to enhanced cell proliferation and differentiation, which are crucial for bone regeneration. Additionally, spheroids are easily scalable, allowing high-throughput screening and personalized medicine applications. They also exhibit better mechanical properties, closely resembling native bone architecture, and offer improved nutrient and oxygen diffusion. Furthermore, their scaffold-free nature reduces the risk of immunogenicity and simplifies the manufacturing process [11].

This review focuses on the current literature regarding bone health, specifically in cell culture models. We assess existing limitations in 2D models and highlight the need for advancements. The emphasis then shifts to the crucial role of 3D cell culture models in transforming bone research, addressing previous shortcomings. The review outlines how this transition enhances our understanding of bone growth, remodeling, and regeneration. It explores the unique advantages of 3D culture systems across developmental stages and contexts of aging. Special attention is given to the impact of 3D models on toxicological and pharmacological bone research, illustrating their transformative role in drug discovery for bone-related diseases. The review also highlights the potential of personalized medicine in bone health, emphasizing how 3D cell culture models enhance our comprehension of bones throughout their lifespan.

## 2. General Advantages of 3D Cell Culture System

The 3D spheroid culture system has evolved as a vital tool in cell biology. It was initially developed to mimic in vivo tissue architecture more accurately than traditional 2D cultures. Over time, various techniques such as hanging drops, rotating bioreactors, and microfluidic platforms have been refined to culture spheroids from diverse cell types. This system facilitates studies in drug screening, disease modeling, and regenerative medicine due to its ability to replicate physiological cell–cell interactions and gradients of nutrients and oxygen. Continuous advancements, including incorporating biomaterials and imaging technologies, further enhance its utility in biomedical research.

Generally, 3D cell culture systems have numerous advantages in various biomedical research and drug development fields. In contrast to 2D cultures, 3D cultures can imitate native cellular architecture, multicellular interactions, and extracellular matrix (ECM) composition. Bone 3D spheroids enhance spatial organization and cell-to-ECM interactions. Unlike the flat monolayer arrangement in 2D cultures, 3D spheroids allow cells to be closely packed into the layers, facilitating increased proximity and interactions among cells. This spatial arrangement enables a better representation of the intricate cell–ECM interactions, capturing the complexity of the natural cellular environment more accurately. Additionally, the freedom of cell movement within the 3D space allows a more dynamic distribution, reflecting a more realistic portrayal of ECM components. Overall, the 3D nature of spheroids provides a more physiologically relevant context, promoting enhanced modeling of ECM components and their interactions compared with traditional 2D cultures. Therefore, 3D systems closely resemble in vivo conditions, enabling researchers to investigate cellular responses and behaviors [12,13,14].

Cells in 3D cultures can interact with neighboring cells in a manner similar to the complexity found in living organisms. This nuanced multicellular interplay is pivotal for capturing intricate physiological responses. It facilitates the study of fundamental processes, such as cell signaling, differentiation, and tissue development, with a level of authenticity that closely mirrors the in vivo environment. By mimicking the original 3D structure of living organisms, it is possible to gain invaluable insights into how cells behave and adapt, paving the way for precision disease modeling, drug testing, and regenerative medicine development.

In 3D cultures, cell–cell communication plays a pivotal role in shaping gene expression, growth factor signaling, and the development of multicellular structures, offering distinct advantages over 2D systems. The 3D environment better mimics the in vivo conditions, fostering intricate cell interactions. This spatial arrangement facilitates enhanced paracrine signaling and influences gene expression patterns and growth factor responses. Moreover, forming multicellular structures in 3D models better reflects tissue complexity and provides a more physiologically relevant context for studying disease mechanisms and tissue regeneration. The 3D culture system allows nuanced exploration of cellular crosstalk, shedding light on the intricate molecular processes underlying diseases and offering valuable insights for advancing therapeutic strategies and regenerative medicine [8,12]. The ECM, one of the major components of 3D cultures, can be tailored to mimic specific tissues of interest. Given that ECM molecules include matrix proteins, glycoproteins, glycosaminoglycans, proteoglycans, ECM-sequestered growth factors, vascular endothelial growth factors, platelet-derived growth factors, and other secreted proteins [12], this customization allows the study of the effects of various ECM components on cellular behaviors and functions, including adhesion, migration, and differentiation [15,16]. It is particularly valuable for investigating diseases associated with ECM abnormalities, such as fibrosis or cancer metastasis [17].

As highlighted by Booij et al., the advantages of 3D systems provide physiologically relevant platforms for drug testing and toxicology research [18]. Compared with 2D cultures, cells cultured in 3D environments often exhibit behaviors that closely resemble in vivo conditions, rendering them resistant to drug-induced toxicity or prompting different responses. This distinction is crucial for accurately assessing drug efficacy and safety and potentially reducing the likelihood of late-stage failures in drug development. The ability to reliably predict drug behavior and toxicity at an earlier stage enhances cost efficiency for pharmaceutical companies. It contributes to the success of bringing new drugs to the market.

Additionally, 3D systems can revolutionize cancer research by enabling the study of tumor biology and drug responses in a realistic context [18]. Furthermore, 3D systems offer significant advantages in generating tumor spheroids or organoids that faithfully recapitulate the heterogeneity and microenvironment of actual tumors [19]. Unlike traditional 2D cell cultures, 3D systems allow the creation of 3D structures that more accurately mimic the complexity of actual tumor tissues, capturing the diverse cell types, spatial arrangements, and interactions in tumors and providing researchers with a more physiologically relevant model for studying cancer biology and drug responses. Tumor spheroids or organoids generated in 3D systems better represent in vivo conditions and offer valuable insights into the intricacies of tumor development, progression, and response to therapeutic interventions. This enhanced modeling fidelity contributes to more effective preclinical studies and holds great promise for advancing cancer research and treatment strategies.

Collectively, 3D cell culture systems offer a physiologically accurate representation of tissue architecture and cell–cell interactions, which may play a central role in biomedical and pharmacological research. Moreover, 3D systems accelerate studies of tissue biology, elucidate disease processes, evaluate drug responses, and advance personalized medicine. The enhanced relevance of 3D culture fosters a deeper understanding of complex cellular behaviors. It facilitates the development of targeted therapies, paving the way for effective treatments and transformative innovations in healthcare.

## 3. The Purpose and Advantages of Using 3D Culture in Bone Research

Bone homeostasis is maintained by two cell types: osteoblasts and osteoclasts. Osteoblasts are responsible for bone formation and the synthesis of the bone matrix, whereas osteoclasts, which specialize in bone resorption, break down old or damaged bones [20,21]. This dynamic process, known as bone remodeling, is influenced by various factors such as hormones, mechanical stress, and growth [22,23]. In 3D culture models, mimicking normal bone physiology is crucial for studying bone-related diseases, drug testing, and tissue engineering. Creating a 3D culture model provides a physiologically relevant platform that better represents the complexity of bone tissue and its interactions, thereby contributing to advancements in bone research and therapeutic development. Incorporating osteoblasts, osteoclasts, and other relevant cells into these systems provides a closer representation of bone physiology and insights into bone remodeling, disease mechanisms, and therapeutic interventions.

### 3.1. General Physiology of the Bone

Bone homeostasis involves the dynamic interplay between osteoblasts and osteoclasts. The coordinated activities of these two cell types ensure the continuous renewal and adaptation of bone tissue, enabling it to serve vital functions in the body, including structural support, protection of organs, mineral storage, and blood cell production.

Osteoblasts are essential for developing and maintaining bone tissue [24,25]. They are primarily involved in bone deposition and bone matrix synthesis, including type I collagen, osteocalcin (OCN), osteopontin (OPN), bone sialoprotein, bone morphogenetic proteins (BMPs), osteoprotegerin (OPG), and receptor activator of nuclear factor kappa-B ligand (RANKL) [26,27]. Osteoblasts release vesicles containing calcium and phosphate ions into the matrix, forming hydroxyapatite crystals that impart hardness and rigidity to bones [28]. This process is essential for maintaining normal blood calcium levels. It involves the coordinated activity of all bone cells, ensuring that osteoblasts can create a calcium- and phosphorus-rich matrix and guarantees adequate bone hardness and functionality [22]. Osteoblasts add new tissue to the bone surface during development and growth, contributing to longitudinal and radial growth [29,30]. Osteocytes are mature bone cells embedded in the bone matrix that originate from osteoblasts and act as mechanosensors and orchestrators of the bone remodeling process [31,32]. Osteocytes initiate four distinct pathways, including formation modeling, targeted remodeling that occurs with heightened mechanical loading, resorption modeling, and disuse-mediated remodeling that occurs in response to changing mechanical demands and regulates whole-bone stiffness, to facilitate the adaptation of bone to changes in the mechanical environment [33]. These pathways regulate whole-bone stiffness in response to changing mechanical demands.

Osteoclasts break down and resorb bone tissue and are crucial for bone remodeling and maintaining calcium homeostasis [21,34]. Osteoclast activity is regulated by several hormones, including parathyroid hormone (PTH) and calcitonin. PTH, produced by the parathyroid glands, stimulates osteoclasts to resorb bone and release calcium into the bloodstream. This occurs by increasing RANKL expression in osteoblasts, which promotes osteoclast differentiation and activity. In contrast, calcitonin produced by the thyroid gland inhibits osteoclasts; it reduces bone resorption by suppressing osteoclast activity and promoting calcium deposition in bones [35]. Osteoclasts secrete enzymes and acids that dissolve the mineralized matrix and break down collagen fibers within bone tissue. Cathepsin K (CatK) is a major enzyme involved in bone resorption. Additionally, osteoclasts secrete hydrochloric acid to form an acidic environment that promotes the dissolution of hydroxyapatite crystals in the bone matrix [36,37]. CatK and acidic conditions are combined during bone matrix remodeling so that the bone matrix can effectively reabsorb bone tissue. This process releases calcium and phosphate ions into the bloodstream, rendering them suitable for other physiological processes. This cycle helps repair microdamage in the bone, adapt the bone structure to mechanical loads, and replace old or damaged bone tissue with new, healthy bone.

### 3.2. Importance and Advantages of 3D Culture Systems for Bone Study

One of the primary benefits of 3D culture systems in bone research is their ability to enhance cell–cell interactions between osteoblasts and osteoclasts [38]. Generally, 3D spheroids enable the formation of complex cell–cell interactions, providing a more physiologically relevant environment for studying the dynamic balance between bone formation and reabsorption. Osteocytes are the most abundant cells in mature bones and form networks within mineralized substrates. In this context, 3D spheroids enhance the network of osteocytes and enable the spatial organization of bone cells, facilitating their communication within bone-tissue-like structures [39].

Another advantage is matrix mineralization. During mineralization, bone cells deposit minerals into their ECM [40]. The 3D spheroids represent the ECM more accurately, allowing bone cells to deposit minerals in a 3D context, essential for studying the mineralization process. Bones are not flat surfaces but dynamic 3D structures composed of cells embedded within an ECM. Thus, 3D cultures can replicate the extracellular collagen environment, minerals, and growth factors more faithfully than 2D cultures [41]. Additionally, bone physiology relies heavily on the interplay between various cell types, including osteoblasts, osteoclasts, and osteocytes [31]. In 3D cultures, these cells can interact naturally and form multicellular niches, allowing the observation of processes such as bone remodeling, mineralization, and the onset of bone diseases.

Furthermore, the response to mechanical forces is effective in 3D systems [42]. Bones are subjected to mechanical forces in the body, which are crucial for bone health. In addition, mechanical forces are indispensable for maintaining bone homeostasis as they activate osteoblasts and osteoclasts. Mechanical signals activate the dynamic interplay between these cells and help bones adapt to load and mechanical stress changes. Bone formation, reabsorption, and adaptation depend on mechanical signals; therefore, the loss of mechanical stimulation leads to a significantly weakened bone structure, causes osteoporosis, and increases the risk of fractures. Osteocytes convert these signals into biochemical signals that regulate the activity of osteoblasts and osteoclasts when mechanical forces are applied to the bone. Moderate mechanical forces stimulate bone formation, while excessive or insufficient loading can lead to bone resorption [42]. In a research context, 3D culture systems allow controlled mechanical stimuli to mimic the in vivo mechanical environment. To control mechanical stimulation in the 3D culture of bone cells, a combination of specialized equipment, biomaterial design, and sophisticated monitoring techniques can be used to create an environment that closely mimics the physiological conditions of bone tissue [43]. Therefore, to investigate bone physiology from a mechanobiological perspective, it is essential to understand how bone cells respond to mechanical signals. These insights can guide strategies for enhancing bone regeneration and repair.

Additionally, 3D culture supports the osteogenic differentiation of mesenchymal stem cells (MSC) into osteoblasts more effectively than 2D culture [44]. By incorporating factors such as enhanced cell–cell interactions, improved cell-to-ECM interactions, spatial organization, mechanical stimulation, sustained release of growth factors, and efficient nutrient supply, 3D cultures offer advantages that contribute to a more effective promotion of osteogenic differentiation than 2D cultures.

Modeling bone diseases such as osteoporosis, in which the imbalance between bone formation and reabsorption leads to decreased bone density, is easier in 3D than in 2D spheroids [45], as 3D spheroids provide a more realistic platform for investigating the cellular and molecular mechanisms underlying bone pathologies. Additionally, drug screening and therapeutic effects can be studied using bone 3D spheroids. Osteocytes in 3D spheroids are valuable tools for drug screening because they respond to drugs and therapeutic agents in a manner similar to under in vivo conditions [46]. This supports the need to evaluate the efficacy of drugs affecting osteoblast behavior, mineralization, and overall tissue integrity in a more relevant context.

**Figure 1:**
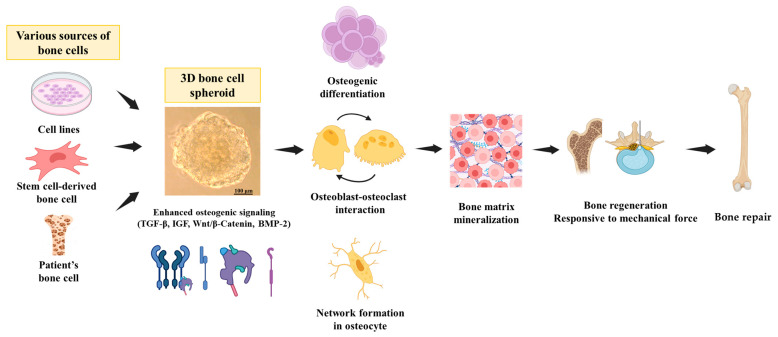
The development of three-dimensional (3D) spheroid bone models for toxicological and pharmacological assessment represents a groundbreaking leap in research. Numerous methodologies have been proposed to craft these 3D spheroid bone models, emphasizing unparalleled advantages. The intricacies of the bone-specific microenvironment within the spheroids are magnified through exhaustive analysis, encompassing critical factors such as cell viability, extracellular matrix (ECM) composition, biomechanical properties, and the integration of osteogenic signaling pathways. This convergence results in an unprecedented enhancement of osteogenic differentiation and the establishment of a fortified osteoblast–osteocyte network. Consequently, the regulation of bone matrix mineralization is significantly refined, while mechanical stimuli exhibit unparalleled efficacy within the bone 3D spheroid system. This perspective can encapsulate the multifaceted essence of bone development and functionality within a meticulously simulated 3D environment, elevating the discernment and applicability of toxicological and pharmacological assessments.

Recently, bone studies using 3D culture systems have become instrumental in advancing regenerative medicine. Researchers can engineer tissue constructs using 3D scaffolds and stem cells to offer promising solutions for bone repair and replacement [47,48]. These tissue-engineered constructs can be tailored to match a patient’s anatomy, reduce graft rejection risk, and improve the overall success rate of bone graft implantation. Furthermore, 3D cultures are also instrumental in modeling bone diseases ranging from rare genetic disorders to common inflammatory conditions such as osteoarthritis [49]. Patient-derived stem cells can be incorporated into these systems to create personalized bone models [50,51], enabling researchers to study disease mechanisms, test potential treatments, and develop patient-specific therapies. This approach can potentially revolutionize the field of precision medicine for bone disorders. It suggests that the bone 3D spheroid system, summarized in Figure 1, provides great opportunities as a versatile tool, as its enhanced bone-specific properties make it valuable not only for studies of bone tissue regeneration and proliferation but also for toxicological and pharmacological assessments.

In conclusion, the use of 3D spheroids in bone studies benefits our knowledge of bone cells, especially by providing a physiologically relevant microenvironment that can reproduce cell–cell interactions, ECM mimicry, spatial organization, and response to mechanical forces. These functions improve the accuracy and applicability of bone cell studies and contribute to a deeper understanding of bone biology and pathology.

### 3.3. Molecular Similarities between 3D Culture Systems and the Bone Environment

In the study of bone-specific proteins in 3D cultures, the focus is often on recapitulating the complex microenvironment of the bone tissue. Here, we outline the key aspects of bone-specific protein expression in 3D cultures. In 3D cultures, the ECM is crucial in providing structural support and biochemical cues. For bone-specific protein expression, the ECM should mimic the composition of the bone matrix, comprising collagen 1 (Col 1), OPN, and OCN, as well as many other factors, as described in Section 3.1.

Collagen is the most abundant protein in the bone matrix. It plays an essential role in the early regulation of bone tissue formation and is an early marker of osteoblast differentiation; this is necessary for the subsequent expression of bone markers [52]. Upregulation of Col 1, fibronectin 1, and laminin expression was observed in 3D spheroids compared with that in 2D monolayer cultures, and all three molecules were involved in increasing survival, proliferation, paracrine effects, and stem cell selection and enrichment in MSCs [53]. Col V and Col VI were also highly expressed in 3D cultures using MSCs, enhancing their proliferation [54]. These results imply that the increased expression of MSC-specific markers in 3D spheroid cultures could contribute to the expression of specific ECM components, including Col I, V, and VI, fibronectin, and laminin.

In 2012, Nguyen and colleagues used a mixed scaffold with collagen-containing 3D materials to study the effects of 3D cell cultures on the differentiation of human MSCs from undefined sources [55]. In their study, 3D cultured cells differentiated from MSCs expressed OCN and OPN at similar levels in bone tissue. Additionally, the level of calcification was higher in differentiated cells than in 2D cultures. This result indicates that 3D scaffolds significantly increased the expression of osteoblastic genes in stem cells as well as the formation of bone minerals. In support of this result, Gurumurthy et al. reported that normalized OCN production was higher in 3D spheroids, while 2D spheroids had no noticeable OCN production [56], suggesting that 3D spheroid cultures may serve as an alternative to 2D cultures for bone tissue research by providing a better microenvironment for the enhanced cellular functions and interactions.

Bone morphogenetic protein (BMP) is a signaling molecule that plays a crucial role in osteogenesis. BMP treatment induces the differentiation of MSCs into osteoblasts. The application of BMP-2 promotes in vitro mineralization and osteogenesis of bone marrow stem cells (BMSCs) in type 2 diabetic mice through the Wnt signaling pathway [57]. BMP-2 binds to its cell surface receptors. Upon binding to the BMP receptor, the receptors undergo conformational changes and initiate intracellular signaling cascades such as Smad1/5/8. BMP-2-induced Smad signaling directly activates the expression of osteogenesis-related genes such as RUNX2, Osterix, and ALP. The 3D system activates BMP-2 signaling and osteogenic differentiation by providing a more physiologically relevant microenvironment [58,59,60,61]. These studies support the idea that the expression of BMP-2 in spheroids stimulates stem cell differentiation. Moreover, BMP-2 was significantly upregulated in 3D spheroid MSCs in a time-dependent manner, indicating that transplantation of 3D spheroids in regeneration therapy contributes to a rapid regeneration process, including new bone formation [58,59].

One of the factors contributing to poor osteogenic regeneration in osteoporosis is the increased cellular senescence of MSCs. While studying the effects of 3D cell culture on cellular aging is a relatively new field, some evidence suggests that the proliferation of cells in 3D cultures may slow cellular senescence [3]. A recent comparative study of cellular senescence from human-derived MSCs cultured in 2D and 3D commercial polysaccharide hydrogel revealed that the level of senescence-specific β-galactosidase was significantly decreased in the 3D samples. Moreover, higher telomerase activity and greater telomere length were observed in MSCs cultured in 3D and were accompanied by a higher osteogenic differentiation potential [62]. This suggests that 3D cultures may reduce cellular senescence in MSCs, thereby promoting the ability to obtain clinically sufficient cell numbers in a shorter duration.

Moreover, the interaction between calcium and phosphate was upregulated in bone 3D spheroids [63]. Calcium and phosphate are essential minerals involved in various cellular processes, including the mineralization and deposition of minerals such as hydroxyapatite [64]. This process is critical for forming and maintaining bones and other mineralized tissues and positively affects mineralization dynamics. The presence of ECM in bone 3D spheroids can affect how calcium and phosphate interact with the matrix.

## 4. Application of Bone Differentiation in 3D Culture Systems

Bone differentiation has long been an important topic in regenerative medicine and bone biology. By providing a physiologically relevant microenvironment, 3D bone culture systems offer a platform for faithfully investigating the dynamic processes involved in bone differentiation [65,66]. In these systems, cells are grown within 3D matrices that can be engineered to imitate the ECM of bone tissue, thereby offering structural support and biochemical cues. Additionally, 3D culture systems allow researchers to apply mechanical stimuli, such as shear forces and fluid flow, to replicate the mechanical cues experienced by bone cells in vivo. This not only influences osteogenic differentiation but also enhances the development of bone tissue constructs [67,68,69].

### 4.1. Studies Showcasing Successful Bone Differentiation Using 3D Culture Models

Bone is a dynamic living tissue that plays a key role in the body by providing structural support, protecting vital organs, and participating in mineral homeostasis [29]. Remodeling occurs continuously through the coordinated action of osteoblasts and osteoclasts for bone recovery and formation [20]. Understanding and controlling bone differentiation processes is important not only to treat skeletal diseases, because the skeletal system involves a continuous cycle of bone resorption and bone formation, but also to increase the success of bone transplantation, implants, and tissue regeneration.

PubMed, ScienceDirect and Scopus were utilized to conduct this review, employing specific keywords: “3D cell culture”, “bone”, “spheroid”, and “bone regeneration”. The search was conducted using independent search strategies, incorporating Basic Boolean operators such as “OR” and “AND” without any restrictions on the year of publication. We have included a list in Table 1 outlining whether cell aggregates, primarily targeted at forming spheroids, were generated through the various 3D cell culture techniques focused on bone tissue regeneration. In this thorough overview, we consolidated findings from 24 studies examining both in vivo and in vitro bone research.

Table 1 summarizes the search for new strategies to optimally regenerate damaged structures, owing to the continuous impact of multiple etiology-induced bone tissue. Therefore, continuous optimization of strategies is essential for analyzing the biological behavior of 3D spheroids in terms of proliferation, survival, and differentiation, confirming that 3D spheroids have far-reaching characteristics superior to those of 2D cultures. MSCs are the most widely used stem cells for 3D models because they play multiple roles in osteogenic systems. MSCs can self-renew or induce growth factors to support the expression of genes associated with ECM formation, calcium content, and alkaline phosphatase (ALP), a marker of initial expression during mineralization. MSCs directly differentiate into osteoblasts and secrete interferon-γ to facilitate osteoblast lineage differentiation [90]. Owing to the renewable nature of MSCs, some studies involve the identification and analysis of accessible sources to provide a clear and concise update on other methodologies used for the analysis of 3D spheroids. The application of 3D spheroids in bone histology will provide more utilization value in the future.

In some studies, BMP-2 has been used to increase the mRNA and protein expression of osteogenesis-related genes. BMP-2 enhanced osteogenic differentiation, stimulated mineralization, and promoted context-dependent cell proliferation, angiogenesis, and vasculogenesis. In addition, BMP-2 stimulated ECM remodeling and chondrogenic effects [58,59]. It has been reported that 3D spheroids activate a number of associated signaling pathways, directly affecting stem cell retention or upregulation [91,92], suggesting the need for alternative cellular characteristics that can be easily expanded and prepared, especially in older patients with metabolic disorders, and that bone 3D systems can be used to manage key molecular mechanisms involved in bone formation.

### 4.2. Highlighting Key Factors and Signaling Pathways Involved in Bone Homeostasis of 3D Spheroids

Studying bone homeostasis using 3D spheroids involves various key factors and signaling pathways. Cell types and the ECM composition are crucial [8], and 3D conditions are required to include related cell types such as osteoblasts, osteoclasts, and osteoclasts, as well as other supporting cells such as MSCs and endothelial cells. The composition should be similar to the cell diversity observed in bone tissue. It is also necessary to mimic the native bone ECM, including collagen, fibronectin, and hydroxyapatite, because it provides structural support and biochemical cues for cell adhesion, migration, and differentiation.

Mechanical stimulation of bone 3D spheroids is essential as it influences cell behavior, matrix production, and bone remodeling; this can be achieved using specialized bioreactors or culture systems [93]. Maintaining calcium and phosphate homeostasis is also important. Appropriate levels of calcium and phosphate, which play crucial roles in bone formation and maintenance, should be maintained to support mineralization [94]. Vascularization of bone 3D culture is also required, as a vascular component is necessary to simulate bone blood supply, nutrient delivery, and waste removal, influencing bone health [95,96].

Cell signaling pathways, growth factors, and cytokines such as bone BMPs, transforming growth factor-beta (TGF-β), insulin-like growth factor (IGF), and RANKL are crucial as they are involved in bone homeostasis. Understanding these signaling pathways is important for maintaining bone health and homeostasis.

In bone 3D spheroids, TGF-β plays a pivotal role in regulating bone homeostasis, as it orchestrates cellular activities by promoting osteoblast differentiation and ECM synthesis, which is essential for bone formation. TGF-β stimulates MSCs to differentiate into osteogenic lineages, thereby fostering the development of mineralized bone tissue within spheroids [97,98]. Moreover, TGF-β modulates interactions between osteoblasts and osteoclasts, influencing bone remodeling processes. These functions of TGF-β in bone 3D spheroids highlight its significance in orchestrating the intricate balance between bone formation and resorption, which is crucial for maintaining skeletal integrity and function.

IGF plays an important role in bone metabolism [99]. It stimulates the synthesis of bone matrix components by promoting osteoblast proliferation and differentiation, enhancing the formation of mineralized bone tissue within the 3D spheroids. Additionally, IGF affects osteoclast activity; its presence in 3D spheroids promotes a balanced bone remodeling cycle, ensuring adequate bone formation [100]. IGF contributes to maintaining bone structure and function within bone 3D spheroids by regulating osteoblast and bone cell functions.

With respect to the signaling pathway, the canonical Wnt/β-catenin pathway stimulates osteoblast activity and promotes bone formation [101,102]. Wnt ligands bind to osteoblast receptors, initiating a cascade of events that ultimately drive bone mineralization. Additionally, the non-canonical Wnt/β-catenin pathway is activated by mechanical stimuli to promote osteogenesis [103]. Research has shown that 3D spheroids of mesenchymal stem/stromal cells promote bone formation by activating the Wnt/β-catenin pathway [104,105]. This pathway was activated in 3D spheroids with enhanced expression of stemness-associated genes, such as Nanog, Oct4, Klf4, and Sox 2 [75,106]. The 3D spheroids quickly promoted osteoblast production and new bone formation through the synergistic activation of the Wnt/β-catenin pathway in vitro.

Another significant signaling pathway is the RANK/RANKL/OPG pathway, which is tightly regulated to maintain the balance between bone formation and resorption. Osteoblasts within the 3D spheroid model continue to produce RANKL as they would in a physiological bone microenvironment. Osteoclast precursors or osteoclast-like cells are incorporated into spheroids, and the surfaces of these osteoclast precursors express the RANK receptor. Within 3D spheroids, the interaction between RANKL and RANK on osteoclasts is more controlled and spatially defined than in 2D cultures [107]. Estrogen, a female hormone, regulates osteoclast differentiation by inhibiting RANKL signaling [108]. Estrogen receptors are present on osteoblasts, and estrogen signaling influences the production of RANKL and OPG. Estrogen downregulates RANKL expression while simultaneously upregulating OPG production, which inhibits RANKL [109].

However, the role of the RANK/RANKL/OPG pathway in osteogenesis in bone 3D spheroids is not fully understood. Recently, this mechanism has been shown to play an important role in aging, hormonal imbalance, and malnutrition [110].

Vitamin D stimulates the absorption of calcium and phosphate in the intestine. Low serum vitamin D levels cause secondary hyperparathyroidism, which increases bone resorption, decreases bone density, and increases the incidence of fractures [111,112]. Vitamin D significantly enhances the expression of RUNX2, OCN, and COL1A1 genes in stem cell spheroids [113]. Schröder et al. reported that vitamin D accelerates bone stiffness [114], but this finding requires confirmation in 3D osteospheres containing osteoblasts and osteoclasts. Lee et al. also reported that vitamin D significantly increased the expression of RUNX2, OCN, and COL1A1 genes in stem cell spheroids. Therefore, it is possible to investigate how an excess or deficiency of vitamin D affects bone structure and function in the spheroid system.

While 3D cell culture studies pose methodological challenges, advancements in various fields and cutting-edge research have identified key factors for achieving optimal bone 3D spheroid formation in which cells begin to attach to each other and initiate the cell–cell signaling process to obtain an unmodified spheroid form with strong osteogenic potential [5].

## 5. Application of 3D Culture Systems for Toxicological and Pharmacological Evaluation in Bone Research

Toxicological evaluation within 3D culture systems is a fundamental technique to ensure the safety of potential drugs or compounds, particularly in the context of bone tissue [8,12,115]. In 3D cultures, bone cells can be exposed to different concentrations of substances, allowing researchers to comprehensively assess their effects. Within this 3D framework, researchers can scrutinize various parameters, such as cytotoxicity and genotoxicity, and other potential adverse effects on bone cells. Overall, the results offer insights into the safety profiles of drugs and help identify any side effects or detrimental consequences on bone tissue early in the developmental process. This method is a critical tool for ensuring the safety and efficacy of pharmaceuticals designed for bone-related conditions and significantly contributes to the overall success of drug development.

Conversely, pharmacological evaluation focuses on understanding how drugs or therapeutic agents affect bone health and function [12,116]. Studies have used 3D culture systems to investigate the effects of different drugs on bone cell proliferation, differentiation, mineralization, and other vital processes. This allows screening of potential drug candidates for their efficacy in treating bone-related disorders such as osteoporosis, fractures, or bone cancers. Furthermore, 3D systems help understand the interaction between drugs and bone ECM, providing insights into how drugs are delivered and retained within the bone tissue [3,8].

### 5.1. Advantages of 3D Cultures of Bone Models in Toxicological and Pharmacological Studies

Applying 3D cultures in bone research has greatly improved our understanding of bone biology and the effectiveness of various drugs and compounds for bone health [117]. This section explores the benefits of using 3D spheroid bone models in toxicological and pharmacological studies. First, by screening potential bone-targeted drugs or therapeutic agents, researchers can assess drug absorption, distribution, metabolism, and excretion within a 3D spheroid bone model, which are critical parameters of drug development. This approach aims to minimize failures during the late stages of drug development [115,118]. Other benefits have been utilized in research on complex interactions [119]. As bone health is affected by various factors, including hormones, growth factors, and mechanical forces [120], 3D spheroid bone models enable the investigation of complex interactions between these factors and their effects on bone tissue. Additionally, 3D spheroid bone models can simulate various pathological conditions, such as osteoporosis and bone metastasis, and can manipulate culture conditions to evaluate the effectiveness of potential drug treatments in a controlled environment, making them advantageous in pharmacological studies.

Given the increasing older population, the long-term effects of drugs or toxins are of great concern in many toxicological and pharmacological studies [121]. The 3D spheroid bone model enables the long-term evaluation of chronic exposure to drugs or toxins, which is important for understanding how compounds can affect bone health over time and identifying potential cumulative effects that may not appear in short-term studies. Conducting long-term evaluations under 2D conditions is challenging due to their inherent limitations. Older persons often exhibit unique responses to medications and toxins, which can be better understood in a more realistic 3D environment that mimics the intricacies of aging bones or tissues. Moreover, 2D cultures struggle to maintain cell viability over extended periods [91], making them unsuitable for chronic exposure studies. Consequently, 3D models are essential for accurately assessing the long-term impacts on older people’s health.

Using 3D spheroids unlocks the potential for personalized medical applications [122]. Such 3D spheroid bone models can be generated from patient-derived cells, enabling the development of personalized medical approaches. Moreover, bone 3D spheroids can mimic individual bone tissues, enabling drugs or treatments tailored to specific patient needs. This personalized approach is likely to revolutionize the treatment of bone-related diseases. In 2021, Abraham et al. reported that bone organoids demonstrated osteogenesis and microvascularization, while cartilage organoids exhibited cartilage development and maturation [123]. The culture served as a model for inflammatory diseases and facilitated the testing of adenosine (A2A) receptor agonists as potential therapeutic agents. To assess the efficacy of organoids as a biological model for drug testing, the group investigated the impact of A2AR stimulation on skeletogenic differentiation and maturation. In 2023, Febre et al. developed a microfluidic protocol to expose the spheroids of Ewing sarcoma, a type of bone or soft tissue cancer often found in the body’s long bones, to drug combinations. Image analysis provided data on hundreds of individual spheroids per experimental run [124]. In this study, sequential combination treatment of Ewing sarcoma with etoposide administered 24 h before cisplatin resulted in an amplified synergistic effect. Hao et al. utilized a bone-on-chip model to examine the bone metastasis of breast cancer. This system effectively simulated the interaction of cancer cells with the bone matrix, allowing observation of the unique characteristics of breast cancer colonization [125]. Reflecting on these findings, it is considered that 3D bone spheroids could be employed alongside more high-throughput systems to test drugs and agents during genetic or pharmacological assays [126]. Overall, incorporating these discoveries, bone organoids show promise as powerful tools in testing systems for disease modeling and drug development.

### 5.2. Benefits for Toxicological and Pharmacological Bone Research in 3D Culture Systems

A comparison of 2D and 3D culture systems for the study of bone toxicity and pharmacology revealed distinct pros and cons. In 2D culture systems, cells are grown on flat surfaces that are easier to set up and maintain, making them cost-effective and amenable to high-throughput screening (HTS). This allows for better control over the experimental conditions and facilitates standardization. However, these systems lack physiological relevance and fail to replicate the intricate 3D architecture of the bone tissue. Consequently, drug responses and toxicological outcomes may differ significantly depending on the in vivo conditions. Cells in 2D cultures also lack the spatial organization and intercellular interactions that are present in 3D systems, which can lead to incomplete or misleading results. Additionally, drug penetration in 2D systems may differ markedly from that in vivo, affecting pharmacokinetics.

In contrast, 3D systems provide a physiologically relevant environment for studying bone tissues. However, this is technically challenging, requires specialized equipment, agents and expertise, and exhibits high experimental variability owing to its complexity. Moreover, although some spheroids have advantages in compliance with HTS, the use of 3D systems remains challenging, making them less suitable for rapid compound screening [115].

For more comprehensive investigation, 3D and 2D systems are often selected based on research goals while considering the tradeoffs between physiological relevance, complexity, experimental control, and resource constraints. A combination of 2D and 3D cultures is frequently employed to complement the strengths and weaknesses in drug development and toxicity testing. Table 2 summarizes the advantages and disadvantages of 3D and 2D systems in toxicological and pharmacological bone studies.

As summarized in Table 2, the choice between 3D and 2D cultures in toxicological and pharmacological studies related to bone research significantly affects the experimental outcomes. While 2D cultures provide a simplified and cost-effective platform, 3D cultures better mimic the in vivo microenvironment, enhancing cell–cell, and cell–matrix interactions. Bone 3D models offer improved physiological relevance, allowing more accurate assessments of drug toxicity and efficacy in bone-related studies. The spatial organization in 3D cultures better represents bone architecture and promotes bone cell differentiation and bone-specific responses. Consequently, bone 3D cultures are valuable for predicting complex in vivo responses, making them increasingly essential for advancing drug development for bone-related conditions compared with 2D cultures. This innovative approach has applications in biomedical research and extends beyond regenerative medicine, including essential evaluations in drug and toxicity research.

## 6. Challenges and Future Prospects of 3D Culture Systems in Bone Research

As previously described, bone is composed of multiple cell types as well as a mineralized ECM [79,139], and accurate in vivo recreation of these components is challenging. It is recognized that 3D bone culture systems can be employed to study the molecular mechanisms underlying osteogenesis, screen drug candidates for bone-related disorders, and develop bone-tissue-engineered constructs for bone repair and regeneration. Additionally, the integration of advanced technologies, such as 3D bioprinting and microfluidics, holds promise for further advancing the field of bone differentiation in 3D culture systems [47,140]. In 3D cultures, the diffusion of nutrients and oxygen is limited as the size of the culture increases [141]. This can lead to potential cell death at the core of the spheroids as well as a lack of uniformity in cellular behavior. These challenges must be resolved to proceed with reliable bone research using 3D systems. The future of 3D culture in bone research lies in innovative biomaterials, advanced technologies, and a collaborative approach, promising a deeper understanding of bone biology and improved treatments for bone-related diseases.

### 6.1. Challenges Associated with Bone 3D Spheroid Systems in Toxicological and Pharmacological Development

Although bone 3D culture systems are accepted as valuable tools in toxicology and pharmacology, they still have some limitations that need to be addressed to improve their usefulness and relevance in research and drug development. A major limitation is that 3D cultures cannot fully replicate the complex cellular makeup of the bone tissue [142]. Bones are composed of various cell types that function together to maintain bone homeostasis. Most of the existing 3D culture models focus on osteoblasts and exclude other essential cell types, resulting in an incomplete representation of the bone environment. Addressing this limitation requires the development of more sophisticated culture systems that incorporate multiple cell types to enable an accurate simulation of bone physiology.

Another challenge is the lack of vascularization, which is important for nutrient and oxygen supply and removing waste from bone tissue [143]. In vivo, bones are highly vascularized, and this vascular structure plays a crucial role in bone health [95]. The absence of a well-developed vascular system compromises the representation of in vivo conditions, thereby limiting the ability of 3D cultures to accurately imitate complex physiological responses. Research should be conducted to bring approximations closer to the complex interactions between drugs and bone tissue in vivo to bridge these gaps and improve the model’s fidelity. Shifting the research focus toward integrating powerful angiogenic components into 3D bone culture systems is important to overcome this limitation, to allow comprehensive insights into drug behavior, potential toxicity, and overall efficacy.

Recently, a method has been reported to enable rapid fabrication of cell-rich bone models that have been vascularized and interpolated using biomimetic intrafibular collagen mineralization [144]. This system showed that the proposed approach enables extensive and almost homogeneous mineralization of hMSC-containing collagen hydrogels with hyperfine structural tissues and elemental composition similar to human bones. In addition, the method is time-controllable, with a versatility of synthesis that can be initiated and stopped at different time points, and it is highly cytocompatible. Therefore, using this model is thought to have significant implications for the discovery and screening of drugs, regenerative medicine, and studies toward understanding bone physiology and disease.

The significance of the bone-specific ECM is often underestimated in existing models, primarily because of its intricate complexity. The ECM plays a key role by offering structural support and regulating cell behavior within the bone tissue, as established in various studies [12]. However, bone 3D culture systems frequently lack the complexity found in natural ECMs, potentially limiting their ability to accurately replicate cell behaviors, differentiation processes, and drug responses. Improving the biomimicry of ECMs in bone 3D culture models is crucial. These models can be transformed into physiologically relevant platforms for extensive toxicological and pharmacological research. This enhancement holds promise for accurately representing biological interactions and responses, thereby significantly advancing the scientific understanding in these crucial research fields.

The 3D spheroid bone system, while promising for mimicking bone microenvironments, faces challenges in clinical translation due to a lack of standardization in resources and assessment methods. Without established protocols, comparing results between studies becomes difficult, impeding progress in understanding and replicating findings. This inconsistency hampers the reliability and reproducibility required for clinical application. Additionally, without standardized assessment methods, evaluating the efficacy and safety of potential therapies becomes subjective, hindering regulatory approval and adoption in clinical practice. Thus, the absence of standardization poses a significant barrier to effectively translating 3D spheroid bone systems into clinical use.

Lastly, the importance of standardization and reproducibility in bone 3D culture systems cannot be overstated, as emphasized in a previous study [45]. The substantial variability observed in the incubation conditions and protocols across different laboratories poses a significant challenge to achieving consistent and comparable results, ultimately impeding the widespread adoption of these models within the scientific community. Addressing this issue requires the establishment of rigorous standardized protocols, stringent quality control measures, and comprehensive data-reporting guidelines. These measures are indispensable for guaranteeing the robustness and reliability of bone 3D culture systems, enabling accurate comparisons and facilitating broader acceptance and utilization of these models in various research domains, including drug discovery, disease modeling, and regenerative medicine.

### 6.2. Proposing Potential Advancements and Research Directions of Bone 3D Spheroids for Toxicological and Pharmacological Assessments

Despite the abovementioned limitations, bone 3D spheroids represent a cutting-edge approach in toxicology and pharmacology, providing a unique platform for studying the effects of drugs and toxins on bone tissue. They also provide valuable tools for investigating bone-associated diseases and therapeutic interventions. In this section, we propose future developments and research directions for exploiting the potential of bone 3D spheroids.

The use of 3D spheroids is expected to expand to bone disease modeling because spheroids can be developed using cells derived from patients with bone-related diseases such as osteoporosis and bone cancer. This can help explain the underlying mechanisms of disease progression and test the effectiveness of potential treatments [145]. Spheroids can also aid drug screening for bone diseases. Based on these technical developments, bone 3D spheroids can be used in drug HTS to identify novel compounds for treating bone diseases, such as osteoporosis and bone metastases, by evaluating the effects of different drugs on 3D bone culture. Given that this aspect has already been highlighted as a challenge to be addressed (Table 2), resolving this issue is expected to advance drug screening considerably. Additionally, from a toxicological perspective, bone 3D spheroids can be useful tools for assessing the toxicity of medicines and environmental toxins to bone tissue. This evaluation can contribute to the identification of bone-specific toxicological effects that can help ensure the safety of drugs and chemical compounds.

Bone spheroids can also be used to develop bone-regeneration treatments in clinical settings. Culturing spheroids with bone cells derived from patients with osteoporosis can be developed into therapeutics that promote bone healing and regeneration. Additionally, bone 3D spheroids help the study of immune responses to bone-related diseases and treatments by including immune cells within them, which is particularly appropriate in conditions related to bone inflammation, such as rheumatoid arthritis.

Advanced imaging techniques such as micro-computed tomography (micro-CT) and magnetic resonance imaging (MRI) can provide detailed insights into the structure and composition of bone 3D spheroids. Micro-CT visualizes the structure of the bone tissue with high resolution, and MRI clearly shows the soft parts and boundaries of the bone tissue [146,147]. These technologies generate high-resolution 3D images via a noninvasive analysis of blood flow, cell density, and material composition, contributing to an accurate understanding of the shape, size, and density of bone 3D spheroids. These imaging techniques can provide important insights into bone tissue research and medical applications in living organisms.

Meanwhile, a study was conducted on potency of bone regeneration through endochondral ossification (ECO) of human mesenchymal stem cells [148]. It has been shown that MSC-ECM structures form strong bones both in vitro and in vivo through ECO when sequentially exposed to cartilage formation and bone formation cues. In addition, it was confirmed that the ECO protocol significantly improved bone formation by MSC-ECM structures compared with conventional in vitro cultures in bone formation medium alone. The significance of this research is that it is designed to promote direct bone formation, as in youngest ossification (IMO). The developmental information methods reported in these studies represent a robust and effective approach for stem cell-based bone formation, which has been reported to be superior to conventional bone formation induction procedures. Therefore, the linkage with tissue engineering, material science, and many other research aspects through various materials and methods will suggest a variety of methods for future studies related to bone regeneration.

Additionally, tissue engineering using biomaterials designed to imitate the ECM of bone tissue holds significant promise for enhancing the relevance and complexity of bone 3D spheroids. By replicating ECM-like properties in biomaterials, tissue engineers can create a microenvironment that closely mirrors the intricacies of native bone tissue [149,150]. This approach enhances the interaction between cells and the engineered material, promoting better cell adhesion, proliferation, and differentiation within 3D spheroids. These biomimetic constructs’ improved relevance and complexity contribute to a more accurate representation of bone tissue, enabling advanced studies in drug testing, disease modeling, and regenerative medicine. Thus, integrating biomaterials that resemble ECMs in tissue engineering offers advances in engineered bone structures’ sophistication and physiological resemblance.

## 7. Conclusions

The use of bone 3D spheroids in bone research supports significant advancements, particularly in the realms of toxicology and pharmacology, with specific emphasis on elucidating the intricacies of bone differentiation. These leading-edge bone 3D systems are ushering in a paradigm shift, supplanting 2D cultures as the preferred instruments for delving into the nuanced landscape of bone development and its response to pharmaceutical interventions. The 3D culture environment faithfully replicates in vivo conditions, offering insights into the intricate interactions between cells and the ECM. In contrast to the flat and monolayer nature of 2D cultures, 3D cultures yield rich information on molecular signaling and bone tissue development, fostering improved growth and natural communication among bone cells. This unique characteristic facilitates a more accurate assessment of the impact of pharmaceutical compounds on bone differentiation and the associated toxic reactions.

The improved precision achieved using 3D culture systems can revolutionize drug development by ensuring safer and more effective results. This transformative approach promises numerous benefits to patients and healthcare providers. These advancements are anticipated to guide healthcare toward a new era, simultaneously unraveling the enigmatic aspects of bone development, differentiation, and regeneration within the dynamic context of 3D culture systems. The trajectory of bone biology research using 3D spheroids represents a remarkable evolution in toxicology and pharmacology.

## Figures and Tables

**Table 1:** Information from the selected studies related to the 3D spheroid bone system.

Research Category	Highlighted Result
MSC-based bone spheroid research	Mass-produced, quality-controlled MSCs were well integrated into the decorated bones of the lumbar spine [70]. Scaffold-free 3D cultured compact adipose-derived stem cell (ADSC) spheroids survived in vivo and in vitro conditions and promoted bone regeneration [71]. In combination with autologous hMSCs, microtissues can be an innovative alternative to autologous transplantation [72]. Spheroid culture facilitated ectopic mineralization in the composition of the BMSC [73]. Osteogenic differentiation was induced in hMSC/HUVEC spheroids for 10 days to produce bone tissue [74]. The 3D spheroid MSC culture was more stem-like than the 2D monolayer MSC culture, and accelerated osteoblast production and osteogenesis [75]. The 3D spheroid culture allowed the production of hBM mesenchymal cells that retained osteoblast differentiation [76]. Geometric clues and synergistic effects of 3D culture appeared when differentiating them into osteogenic systems [77].
Scaffold 3D-based bone research	Dental pulp–mesenchymal stem cells’ microspheres exhibited osteogenesis, such as human fetal osteoblast microspheres [78]. The 3D-printed poly(L-lactide-co-trimethylene carbonate) scaffolds and modified human platelet lysate hydrogels construct represents a promising scaffold for bone tissue engineering applications [79]. Magnetic levitation culture enabled 3D stable osteoblast spheroids [44]. Oxychip promoted bone formation differentiation of MSCs. In vitro bone formation ability was very similar to that observed in vivo [80]. BMSC resulted in tissue compression rather than growth. Not all mineralized bone-like substrates were included in the bulk microtissue mass [59]. An advanced double 3D bone implant was developed combining a nanostructured bioactive biomaterial and predifferentiated osteogenic microtissues [81]. A bioactive matrix can be utilized for bone regeneration and vascularization, as it can promote spheroid formation and contribute to the formation of vascularized tissue from human whole bone marrow cells [82]. Osteogenic differentiation of 3D microtissues was enhanced by mimicking in vivo conditions more than 2D [58].
Matrigel and methodological application-based bone research	By arranging ADSCs as spheroids, markers of both osteoblastic and angiogenesis can be obtained quickly and spontaneously compared with 2D incubation [83]. Hydroxyapatite and cancellous bone scaffolds exhibited improved cell integration and survival compared with other materials [76]. Spheroids containing HUVECs and human bone marrow stromal cells enhanced bone formation at defect sites in vivo [84]. The presence of fragmented fibers improved the stemness retention of human turbinate mesenchymal stem cells [85]. Polymer matrix-based 3D spheroids of cranial stem cells enhanced multipotency and proliferation while promoting the maintenance of stemness [86]. Co-cultured spheroids composed of primary human osteoblasts and human dermal microvascular endothelial cells represent valuable tools for vascularization in bone tissue engineering [87]. The three-dimensional cultures of hMSC-TERT combined with hydroxyapatite–tricalcium phosphate in osteogenic induction medium replicated many features of in vivo bone formation [88]. The degree of acetylation of chitosan played a crucial role in determining the affinity of human osteoblastic MG-63 cells towards the 3D substrates within the three-dimensional chitosan matrices [89].

**Table 2:** Comparison of 3D and 2D cultures in toxicological and pharmacological bone research.

	Pros	Cons
3D	Physiological relevance: Better mimic the in vivo microenvironment of bone tissue, providing a physiologically relevant platform for studying drug toxicity and response [4,7].	Technical challenges: Establishing and maintaining can be technically challenging and resource-intensive. May require specialized equipment and expertise [8].
	Complexity: Allow the recreation of the three-dimensional structure of bone tissue, including its cellular and matrix components. Complexity can yield insights into interactions [127].	Variability: Variability can be higher due to the complexity of the system, making it harder to control experimental conditions [12].
	Drug penetration: Often exhibit slower drug penetration compared with 2D systems, which can be advantageous for simulating drug distribution and metabolism more accurately [128,129].	High-throughput: High-throughput screening for bone cells is still difficult, making it less suitable for rapid screening of large numbers of compounds [130].
	Tissue engineering: Available tissue engineering applications, where researchers aim to regenerate or repair bone tissue [131].	Lack of vascularization: Lack of vascularization within the engineered bone grafts inhibits osteogenesis and host integration; inhibits the healing of large bone defects [132,133].
	Long-term studies: In many toxicological and pharmacological studies, 3D spheroid bone models can be maintained for extended periods, enabling the assessment of chronic exposure to drugs or toxins [134].	
2D	Simplicity: Easier to set up and maintain the bone cell. Often cost-effective and amenable to high-throughput screening [7].	Limited physiological relevance: Do not accurately represent the three-dimensional architecture and microenvironment of bone tissue. Can lead to discrepancies in drug responses and toxicity compared with in vivo conditions [7,68].
	Control: Easy control over experimental conditions. Easier to standardize experiments and achieve reproducible results [135].	Limited interaction: Lack the spatial organization and intercellular interactions found in 3D systems, potentially leading to incomplete or misleading results [136].
	Drug screening: Well suited for initial drug screening and toxicology studies for bone cells, allowing quick identification of potential candidates for further testing in more complex models [137].	Drug penetration: Drugs may penetrate cells more rapidly than they would in vivo, leading to different pharmacokinetics [138].
